# Principles of Anatomy and Physiology

**Published:** 1911-09

**Authors:** 


**Principles of Anatomy and Physiology; John Aitken,** M.D., Fellow of the Royal College of Surgeons, and the like. Printed for **J. Murray,** London, **1786.** Price not stated. About 350 pages with numerous plates. Bound in calf.

This interesting work has lately been presented to the editor who will gladly show it to anyone who wishes to examine it. The style is brief but clear. Practical, surgical and medical hints abound. Dislocations and fracures are discussed for the various bones. Under the head of teeth are included some very valuable suggestions as to oral hygiene and the operation of transplanting is mentioned as a "very ornamental piece of surgery." We select a few passages at random: "When a person falls on the neck and is stunned or motionless, these vertebrae (1st and 2d) may be supposed to be disturbed, an idea that justifies gentle extension with a view to relieve the spinal marrow from compression." "A small share of the pericardium touches the sternum. . . . so as to render it practicable to open it through a trepaned hole of this bone without wounding the pleurae. May this be done to discharge dropsy there?" "The facial artery is in the way of the extirpation of the maxillary gland or lymphatic ones connected with it, which are often tainted from the cancerous lip and require to be removed along with it." Many similar allusions make us realise the comparatively slight advance of surgery, save in regard to anesthesia and asepsis, since this book was published.

The plates are exceedingly clear, so far as we can judge, accurate, and much better executed than those in the anatomy which we studied a hundred years later.

